# Numerical Study for Blood Flow in Pulmonary Arteries after Repair of Tetralogy of Fallot

**DOI:** 10.1155/2012/198108

**Published:** 2012-12-27

**Authors:** Ming-Jyh Chern, Ming-Ting Wu, Sheau-Wei Her

**Affiliations:** ^1^Department of Mechanical Engineering, National Taiwan University of Science and Technology, Taipei 10607, Taiwan; ^2^Department of Radiology, Kaohsiung Veterans General Hospital, Kaohsiung 81362, Taiwan; ^3^Faculty of Medicine, School of Medicine, National Yang-Ming University, Taipei 112, Taiwan

## Abstract

Pulmonary regurgitation (PR) is a common phenomenon in pulmonary arteries in patients after repair of tetralogy of Fallot (TOF). The regurgitation fraction of left pulmonary artery (LPA) is usually greater than right pulmonary artery (RPA) according to clinic data. It may be related to blood flow in pulmonary arteries. Therefore, understanding hemodynamics in pulmonary arteries helps to comprehend the reason. The aim of this study is to use 3D reconstructed pulmonary artery models from magnetic resonance imaging (MRI) and to use numerical approaches for simulation of flow variations in pulmonary arteries after repair of TOF. From the numerical results, the blood flow is influenced by the bifurcation angles and geometry of pulmonary artery. The regurgitation happens first in LPA after repair of TOF due to the small angle between LPA and main pulmonary artery (MPA). The recirculation region which obstructs forward blood flow to the left lung is found in LPA during acceleration of systole. We also analyze the pressure distribution; the extreme pressure variations are in dilation area of MPA. Numerical data including regurgitation in MPA, LPA, and RPA are compared with phase contrast MR measured data. Good agreements are found between numerical results and measured data.

## 1. Introduction

Tetralogy of Fallot (TOF) is the most common congenital heart disease, accounting for 10% of all common congenital heart diseases. TOF is composed of (1) ventricular septal defect, (2) pulmonary stenosis, (3) right ventricular hypertrophy, and (4) overriding aorta. Surgerical correction is the fundamental treatment. However, the surgical reconstruction of TOF usually complicates with pulmonary regurgitation (PR) phenomena. PR is caused by incomplete close and deformity of the pulmonary valve after surgical reconstruction of the right ventricular outflow track in repaired TOF. It causes complex blood flow in pulmonary arteries, which is related to right ventricular dysfunction. Phase contrast magnetic resonance (PC-MR) technology is an established noninvasive technique to estimate blood flow in the vessels and has been used to investigate the PR phenomena in repaired TOF patients. According to Kang et al.'s [1] PC-MR measurements of repaired TOF, it was found that the regurgitation fraction (RF, i.e., backward flow volume/forward flow volume) of PR in left pulmonary artery (LPA) is higher than right pulmonary artery (RPA) in repaired TOF. The reason causing this discrepancy of RF is still not clear. We hypothesize that the surgically created structure-fluid dynamical interaction is one of the possible reasons. In this study, we utilize numerical modeling to investigate the relation between the structure, fluid dynamic, and PR in repaired TOF.

Ho et al. [2] reported many complications in 21 TOF patients after surgery according to their cardiac MR measurements, for example, severe pulmonary regurgitation (PR), right ventricular dilatation, right ventricular outflow obstruction, ventricular septal defect patch leakage, and arrhythmias. Kang et al. [1] discussed the difference of PR between left and right pulmonary arteries from PC-MRI measurements for 22 patients after repaired TOF surgery. They found that the regurgitant fraction in LPA is greater than that in RPA and MPA in most of repaired TOF patients. Meanwhile, Kang et al. [1] also indicated that the relationship between the regurgitant fraction and fraction of regurgitant flow duration in MPA and RPA is linear. Wu et al. [3] investigated the effect of pulmonary regurgitation on perfusion of the lungs in 43 patients after repaired TOF using PC-MRI. They calculated forward flow volume, backward flow volume, net flow volume, and regurgitation fraction in the left and right pulmonary arteries. Comparison of the perfusion between the left lung (L%) (L% = left lung/left + right lung) was provided in their PC-MRI measurements. In terms of the comparison, they also found that the PR phnomenon of LPA is higher than RPA in repaired TOF patients.

Helbing et al. [4] explored the abnormalities in right ventricular (RV) diastolic function with 19 children after repair of TOF and 12 healthy volunteers by PC-MR technique. They found that right ventricular end-diastolic volume (RVEDV) of repaired TOF patients is larger than a healthy person's. Furthermore, it was revealed that right ventricular ejection fraction of repaired TOF is lower than healthy children. Frigiola et al. [5] demonstrated that RV systolic function in 124 patients with surgically treated TOF is affected by PR. In all patients, right ventricular systolic function of repaired TOF patients is poorer than healthy children. RV isovolumic acceleration is inversely proportional to PR in Frigiola et al.'s results. van den Berg et al. [6] indicated that some risk factors including RV abnormal dilation, long QRS duration, and severe PR are associated with RV dysfunction in terms of PC-MRI measurements and electrocardiogram (ECG) of 59 patients after repair of TOF. Grothoff et al. [7] described that PR is an important factor influencing prolongation of QRS duration in ECG measurements for 67 patients after repair of TOF. They indicated that PR phenomena usually accompany QRS prolongation.

Pulmonary valve replacement (PVR) has been shown to improve ventricular function, stabilize QRS duration, and reduce atrial and ventricular arrhythmias in the following studies. For instance, Vliegen et al. [8] confirmed that PVR can reduce regurgitation and shunting defects in 26 patients after repaired TOF using MRI. Their results showed that the mean preoperative PR was 46 ± 10%. 80% patients had no residual PR after PVR. They also found that right ventricular end-diastolic volume decreased from 305 ± 87 to 210 ± 62 mL and RV end-systolic volume also decreased from 181 ± 67 to 121 ± 58 mL. van Huysduynen et al. [9] showed that PVR can reduce QRS duration from 150 to 140 ms according to their cardiac MR and ECG measurements for 26 patients after repair of TOF. Sung et al. [10] used particle flow visualization experiment to investigate the effects of varying degree of pulmonary valvular stenosis in transparent glass models. Porcine pulmonary arteries and valve were considered to fabricate those glass models. Dilatation existed in MPA and LPA of the glass models. They found that significant secondary flows appear in MPA due to its dilatation. They also observed that strength of the secondary flows in the LPA and RPA increased as the degree of valvular stenosis increased. Their results proved that the geometry of MPA with dilatation and pulmonary valvular stenosis have remarkable effects on the pulmonary artery hemodynamics.

The variation of blood flow with pulmonary regurgitation in pulmonary arteries is an important factor for evaluation of TOF defects. Computational fluid dynamics (CFD) is a useful and noninvasive method to calculate blood flow in arteries. In recent years, CFD is commonly applied to simulate cardiovascular complex flow. It provides numerical solutions for accurate assessment of congenital heart disease treatments. In addition, applications of CFD on simulations of blood flow in pulmonary arteries can be found in several technical papers. For example, Tang et al. [11] used CFD to compare flow phenomena of two various pulmonary artery models, spiral and Lecompte, (nonspiral) which are usually used in arterial switch operation. They explained that the spiral method is better than the Lecompte method in terms of numerical results which reveals that the spiral method has more uniform velocity distribution, wall shear stress, and less power loss ratio than the Lecompte model. Corno and Mickaily-Huber [12] compared two different pulmonary artery models, circular and elliptical models, of distal conduit anastomosis by CFD. Their study proved that the anastomosis cross-sectional area has a great impact on blood flow distribution in pulmonary artery. They suggested that the elliptical anastomosis may be useful to improve the morbidity and degree of distal stenosis at clinical applications. Chern et al. [13] established *in vitro* pulmonary artery models after repair of TOF and observed the flow patterns with varying PR in pulmonary arteries after repair of TOF patients by CFD simulations. They also confirmed that the PR fraction in LPA is higher than in RPA and in LPA. Also, they found that vortices move toward the pulmonary valve during regurgitation in numerical results. The vortex motion may damage the pulmonary valve.

Recently, many studies applied medical images to reconstruct 3D arterial peripheral models for undertaking accurate CFD simulations. Redaelli et al. [14] detailed reconstruction of 3D vascular numerical models from magnetic resonance images and performed CFD simulations using the reconstructed vascular models. Moreover, Tang et al. [15] combined MRI and CFD to analyze blood flow in normal pulmonary arteries under resting and exercise states. They reported wall shear stress (WSS), oscillatory shear index (OSI), and the variation of energy efficiency due to exercise in pulmonary arteries. They found the low mean WSS regions in the distal pulmonary arteries no matter in rest or exercise conditions. High OSI values occurred in those low mean WSS regions and branching locations where swirling flow and separation flow were observed. They discovered the energy efficiency average decrease of 10% between rest and exercise conditions. They concluded that this approach is useful for investigation of the disease development and applications of surgical planning. Das et al. [16] calculated the total energy of the MPA to assess the RV performance between after repaired TOF and normal pulmonary arteries using MRI and CFD. Their results indicated that the repaired TOF RV has lower stroke work than the normal one. They also computed the net energy transferred at the MPA that in normal case has higher net energy than repaired TOF case.

CFD has been applied to surgical planning for pulmonary arteries as well (see [17, 18]). For example, Hsia et al. [19] studied the influence of various inferior vena cava connected to pulmonary arteries on blood flow using CFD simulations. They considered four basic TCPC geometries which were reconstructed from angiographic measurements. They demonstrated that the anastomosis in which extracardiac conduit is connected with left pulmonary artery has the lowest energy loss among four various TCPC cases. They also compared energy dissipation in five graft extracardiac conduits with various diameters (10~30 mm) by CFD. They found that the least energy dissipation occurs in the conduit of diameter 20 mm. They confirmed that the geometry of the surgically created pathway in the TCPC is very important in terms of energy dissipation. Sun et al. [20] investigated the influence of antegrade pulmonary blood flow on bidirectional cavopulmonary anastomosis using CFD. They considered four mean flow rates (0.5 L/min, 1 L/min, 1.5 L/min, and 2 L/min) of MPA. They found that the flow ratio of LPA/RPA increased when the amount of antegrade pulmonary blood flow increased. Increasing antegrade pulmonary blood flow may cause significant different blood flows to two lungs. They also observed that blood flow into LPA from MPA is larger than into RPA because the angle between MPA and LPA is larger than between MPA and RPA.

Furthermore, Pekkan et al. [21] showed that the flow patterns at the normal fetal aortic arch and pulmonary artery, which were obtained by CFD and * in vitro* experiment methods, were similar to each other. They found the swirling flow at the pulmonary artery during the deceleration phase of a cardiac cycle both in the numerical results and in the experimental flow visualization. Wang et al. [22] utilized CFD technique and flow visualization experiments to compare two types of TCPC including intra-atrial connection and extracardiac inferior vena-cava- (IVC-) to-MPA connection. Their results showed that more complex unsteady flow structures occur in intra-atrial connection model in experimental and computational results.

According to those studies mentioned previously, we know the PR always happens in a repaired TOF patient and the blood flow in LPA is less than in RPA from MRI statistical analysis. It is found that the blood flow distribution highly depends on the geometry of vascular vessels. Nevertheless, variation of blood flow in real pulmonary arteries after repair of TOF is not clear. Meanwhile, numerical prediction of blood flow becomes popular in investigating the relationship between hemodynamics and cardiovascular diseases as mentioned in previous paragraphs. Hence, the aim of this study is to observe the blood flow distribution and to discuss the effect of geometry on PR in pulmonary arteries by CFD simulation coupled with 3D reconstructed MRI pulmonary artery models. Furthermore, to find the reason to cause higher PR in LPA than in RPA, numerical simulations are performed to provide distribution of pressure, mass flow rate, regurgitation fraction, and blood flow streamlines in pulmonary arteries after repair of TOF.

## 2. Mathematical Formulae and Numerical Model

In this study, the branching pulmonary artery geometry obtained by MRI is utilized to establish a numerical model in computational fluid dynamics. The pulmonary artery is a Y shape bifurcate vessel including LPA and RPA. Various pulmonary arteries after repair of TOF are measured by MRI and considered to observe regurgitation phenomena. First of all, MRI measurements of pulmonary arteries after repair of TOF are reconstructed as CAD models. Subsequently, mesh generation is undertaken in those reconstructed CAD models. Blood flows are then simulated using a flow solver in those numerical models of pulmonary arteries. Details are demonstrated in the following sections.

### 2.1. MRI and Grid Generation

**Figure 1:**
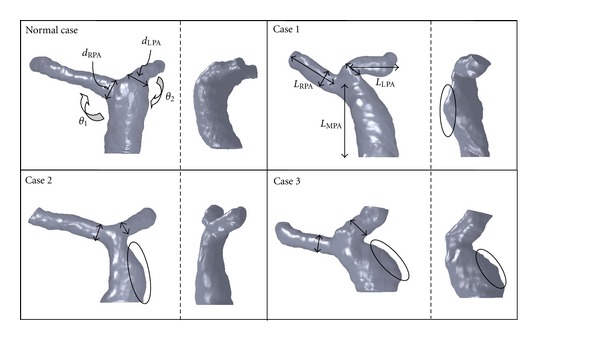


In order to establish a realistic 3D numerical model of pulmonary artery, MRI measurements are utilized to obtain peripheries of pulmonary arteries after repair of TOF and a healthy person. Regions of the branching pulmonary arteries are remarked in slice planes of MR images based on DICOM format. Every slice thickness of Cases  1–3 was 3.6 mm, 4.4 mm, and 4.4 mm, respectively. The normal one was 4 mm. Those 2D remarked regions are connected together be a complete 3D CAD pulmonary artery model. Those features of four pulmonary models are listed in Table 1 and shown in Figure 1. We found that the MPA dilation phenomenon and an acute angle between LPA and MPA appear in all reconstructed CAD models of pulmonary arteries after repair of TOF.

**Figure 2:**
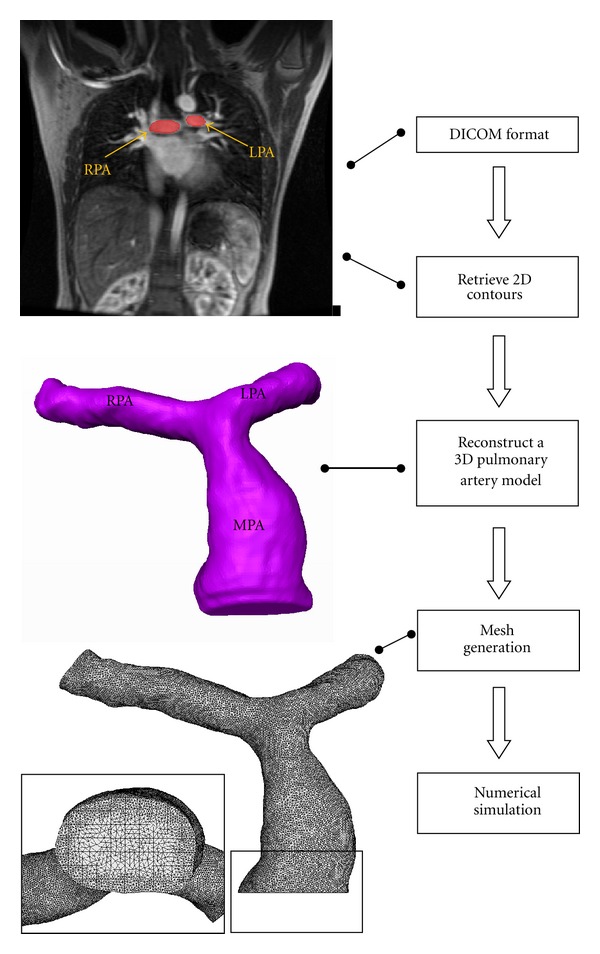
Flow chart of the reconstructed pulmonary artery model.

Subsequently, computational grids are generated inside those reconstructed CAD models using ICEM-CFD software. Since peripheries of the pulmonary arteries are too complicated to generate structured grids, unstructured grids are used in the numerical models.  Figure 2 presents the procedure to reconstruct CAD models from MRI measurements and mesh generation.

### 2.2. Governing Equations

Blood flow obeys mass and momentum conservation. In this study, few assumptions are used to simplify equations for mass and momentum conservation. Mathematical formulae of mass and momentum are denoted as:

continuity equation:

(1) $$\begin{array}{c} \nabla \cdot u=0, \end{array}$$

and momentum equation (Navier-Stokes equation)

(2) $$\begin{array}{c} \frac{\partial u}{\partial t}+\nabla \cdot \left(uu\right)=-\frac{1}{\rho }\nabla P+\nabla \cdot \left(\nu \nabla u\right), \end{array}$$

where **u** represents a velocity vector of flow, *P* is pressure, *t* is time, *ρ* is density of blood, and *ν* is kinematic viscosity of blood. The density of blood, *ρ*, is set as 1060 kg·m^−3^. The dynamic viscosity (*μ*) of blood is set as a constant as 0.004 kg·m^−1^·s^−1^. Although blood is non-Newtonian, viscosity of blood varies with respect to shear rate and vessel diameter. Given that shear rate is greater than 100 s^−1^, the blood viscosity can be regarded as a constant like a Newtonian fluid in accordance with Pedley [23] and Berger and Jou [24]. Because a pulmonary artery is a large vessel, the non-Newtonian effect is not strong. Hence, blood in pulmonary artery is considered as Newtonian fluid for simplifying equations. According to Singh et al. [25], the wall of vessels is regarded as a rigid tube. That is, compliance of pulmonary artery is not considered.

### 2.3. Boundary Conditions

Regurgitation occurs in the pulsatile blood flow of pulmonary arteries after repair of TOF. Regurgitation fraction, RF, is defined as the ratio of backward blood volume to forward blood volume in a cardiac cycle. A health person's RF is usually without regurgitation or very low. High RF indicates that abnormal blood flow behavior occurs in pulmonary arteries. That is, low RF means that most blood flows into lungs to exchange oxygen. RF is defined as

(3) $$\begin{array}{c} \text{RF}=\frac{\text{Backward}\;\text{volume}\;\text{in}\;\text{a}\;\text{cardiac}\;\mathrm{cycle},Q_{b}}{\text{Forward}\;\text{volume}\;\text{in}\;\text{a}\;\text{cardiac}\;\mathrm{cycle},Q_{f}}, \end{array}$$

where *Q*_*b*_ and *Q*_*f*_ refer to backward and forward blood flow volumes in a cardiac cycle, respectively.

**Figure 3:**
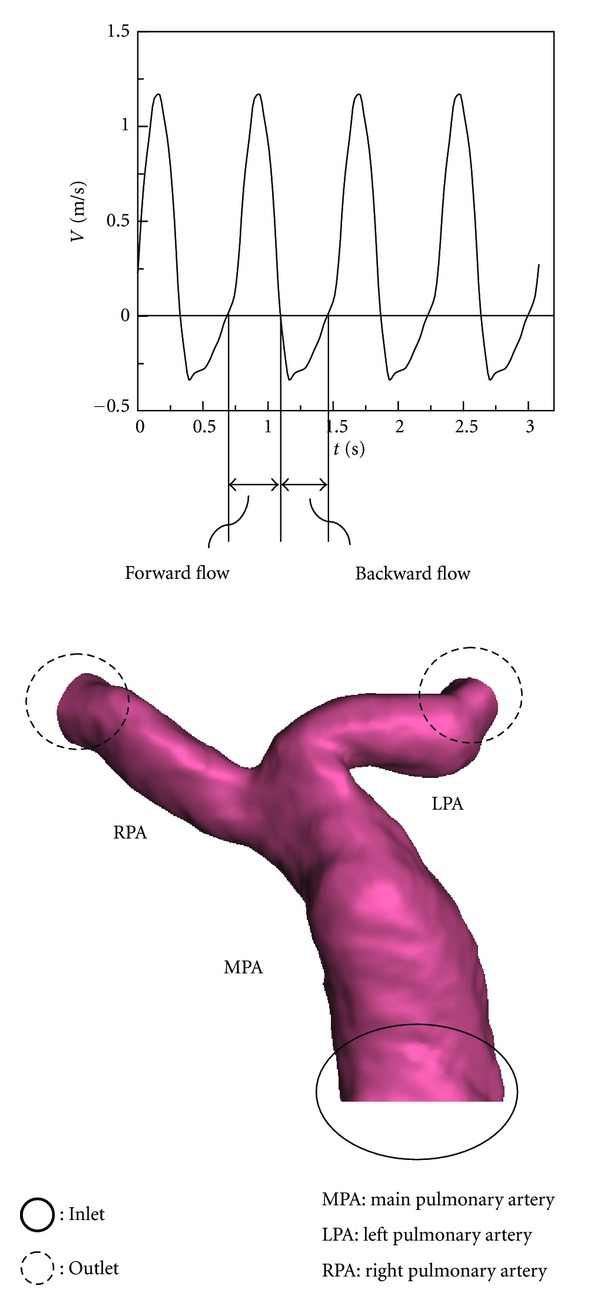
Boundary conditions.

Figure 3 shows boundaries of the established model. To solve those equations, appropriate boundary conditions are required. Blood flow velocity profiles of individual patients were measured by phase contract MRI at the inlet boundary of the MPA entrance. The PC-MR image matrix was 256 pixels × 256 pixels. The image voxel sizes of Cases  1–3 were 0.141 × 0.141, 0.078 × 0.078, and 0.082 × 0.082, respectively. The normal case was 0.0625 × 0.0625. The measured nonuniform flow is provided for the inlet condition. The interpolation method is used to map the inlet velocity profile to inlet grid points. Also, RFs of Cases  1–3 are 0.337, 0.164, and 0.288 as shown in Table 2, respectively.

The atmospheric pressure is imposed at the exits of the RPA and LPA. The wall of vessels is regarded as a rigid object. Nonslip boundary conditions are imposed in vessel wall. That is, velocity is zero in the wall. For the initial conditions of the pulmonary artery, velocity is set as zero.

### 2.4. Parameters Setting

The software, CFD-ACE+, is based on a finite volume method that is used to calculate the blood flow in a pulmonary artery. The finite volume method has been applied to solving the Navier-Stokes equations in many engineering applications. For example, Georgios and Ioannis [26] used the finite volume method in computation of radiative heat transfer. Kabinejadian and Ghista [27] employed the method to solve the NavierStokes equations for simulations of blood flow in a coronary arterial bypass graft. Details of the finite volume method for solving the Navier-Stokes equations can be found in Versteeg and Malalasekera [28]. Computational grids mentioned in Section 2.1 are utilized to discretize the Navier-Stokes equations. As a result, a set of simultaneous algebraic equations for velocity and pressure are obtained and solved implicitly. To observe complex blood flow patterns in pulmonary artery, transient simulations are undertaken. The second-order Crank Nicholson scheme is employed to undertake the time marching procedure. The time step size ranges from 0.01 to 0.015 s. Four cardiac cycles are conducted in each numerical simulation. The total simulation time is different in each case because the duration of a cardiac cycle is different in each person. The mass residual tolerance for numerical solutions is set to 10^−4^ at each time step. The SIMPLEC scheme proposed by van Doormaal and Raithby [29] is used to obtain pressure solutions. The Window-based PC cluster is utilized to perform numerical simulations in this study. The average total calculation time for each case is about 1~2 months.

### 2.5. Grid Independence

**Figure 4:**
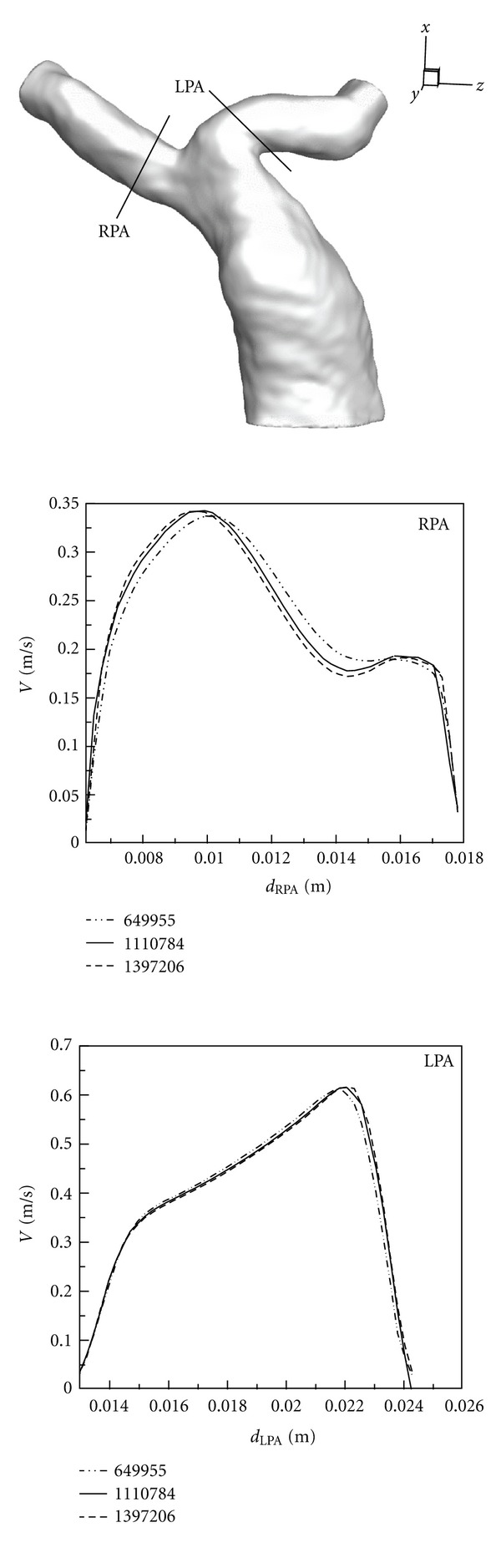
Grid-independence test.

To ensure that the numerical solutions do not vary with the grids, the grid-independence tests must be performed. For example, Figure 4 shows that the velocity profiles obtained by various computational cells are close to each other in Case 1 when the number of the cells exceeds 1110784. Therefore, we adopt 1110784 cells to conduct the following numerical simulations in Case 1. Moreover, we chose the 1168920 and 1035407 cells for Cases 2 and 3 according to results of grid-independence tests, respectively. 1079490 cells are used in the healthy case.

## 3. Results and Discussion

### 3.1. Influences of Bifurcation Angles and Geometry of Pulmonary Artery on Blood Flow

Pulmonary regurgitation is a common phenomenon in patients after repair of TOF, but its effect on pulmonary arteries is not clear. The established numerical pulmonary artery models include one healthy and three pulmonary arteries after repair of TOF. Table 2 shows that Cases  1–3 have smaller angles between LPA and MPA than a healthy one. *θ*_2_ indicating the angle between LPA and MPA is 52°, 83°, and 70° in Cases  1–3, respectively. Nevertheless, *θ*_2_ in a normal pulmonary branch is 112°. On the other hand, angles between RPA and MPA in Cases  1–3 are larger than the normal one. *θ*_1_ indicating the angle between RPA and MPA in Cases  1–3 are 134°, 106°, and 136°, respectively. The angle of the normal one is 125°.

**Figure 5:**
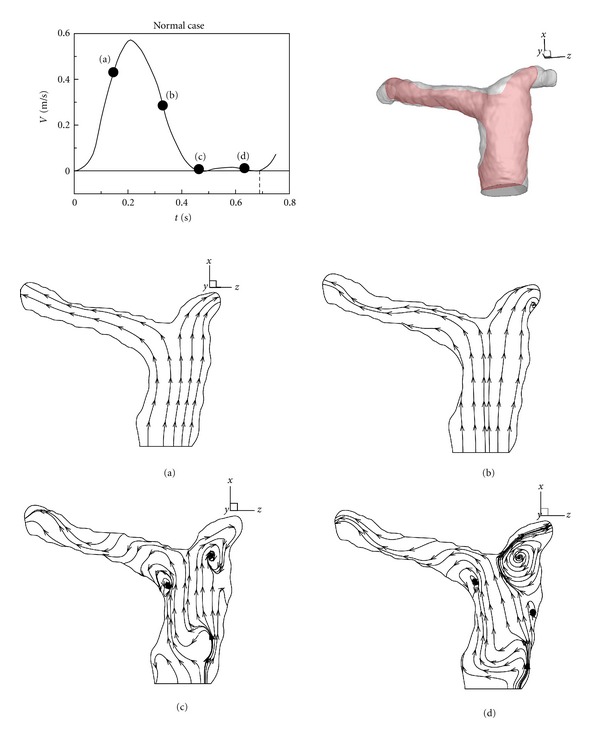
Evolution of streamlines in healthy pulmonary arteries within a cardiac cycle.

Figure 5 describes streamlines of the healthy case in a central vertical sectional plane within a cardiac cycle. There is no regurgitation in healthy pulmonary arteries. Figure 5(a) shows the flow pattern in the acceleration of systole. A smooth forward flow pattern is found in this stage. Subsequently, two recirculations occur in LPA and RPA due to the deceleration of the forward flow in Figure 5(b). Those two vortices grow and stay in entrances of LPA and RPA in diastole of a cardiac cycle as shown in Figures 5(c) and 5(d).

**Figure 6:**
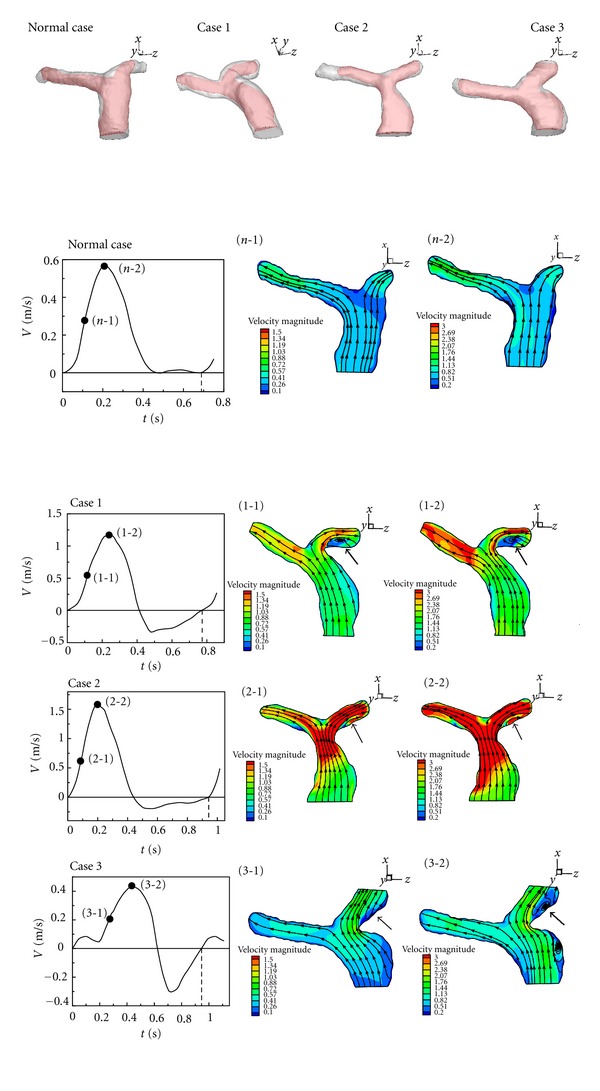
Flow patterns and velocity distribution in midacceleration of systole and maximum in a cardiac cycle.

**Figure 7:**
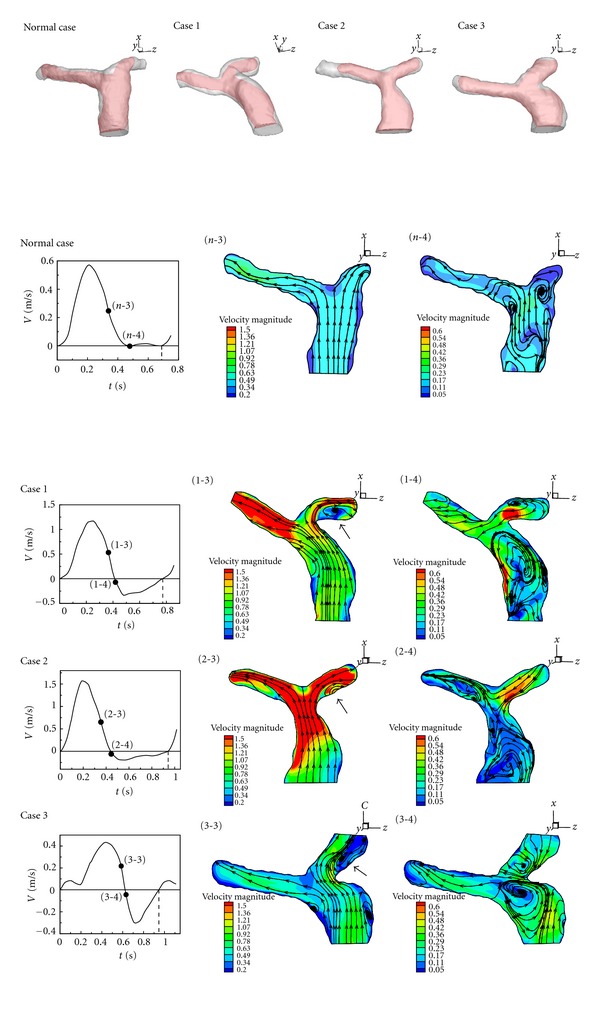
Flow patterns and velocity distribution in deceleration and end of systole in a cardiac cycle.

**Figure 8:**
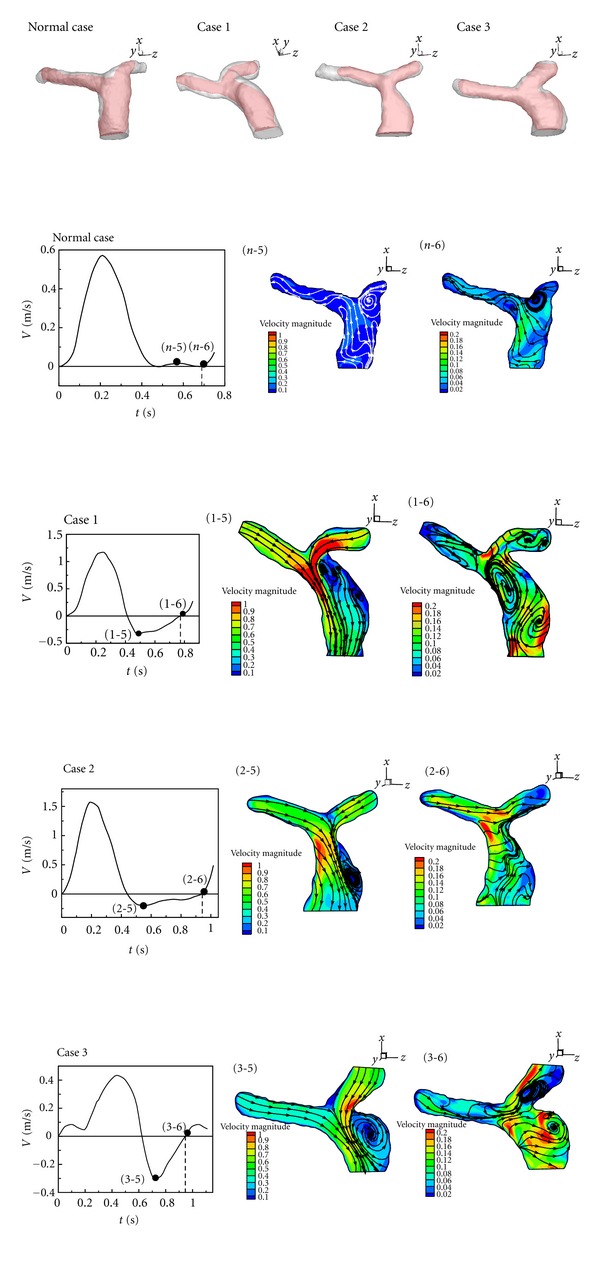
Flow patterns and velocity distribution in minimum of profile and end of diastole in a cardia cycle.

Figure 6 is utilized to compare flow patterns of the healthy one and Cases  1–3 in acceleration of systole and maximum in a cardiac cycle. It is found that a recirculation occurs in LPA in Cases  1–3 but not in the healthy one. It is because *θ*_2_ of Cases  1–3 is smaller than the healthy one. As a result, separation happens in LPA even though the forward flow in MPA is accelerated. The recirculation in LPA reduces the blood flow volumetric rate to the left lung.  Figure 7 (*n*-3), (1-3), (2-3), and (3-3) present streamlines of the healthy one and Cases  1–3 in deceleration of systole in a cardiac cycle. The recirculation bubble in Cases  1–3 grows. As mentioned in the previous paragraph and shown in Figure 7 (*n*-3), there is also a recirculation appearing in the healthy one during deceleration of systole. At the end of systole, very complex flow patterns including several vortices are found in Cases  1–3 as shown in Figure 7 (1-4), (2-4), and (3-4). Subsequently, Figure 8 reveals flow patterns of the healthy one and Cases  1–3 in diastole of a cardiac cycle. Regurgitation happens in MPA of Cases  1–3. Obvious dilations of Cases 2 and 3 are found in MPA. During regurgitation in MPA, a strong vortex is formed in dilation of MPA of Cases 2 and 3. Nevertheless, there are two vortices appearing in MPA of Case 1. No vortex is found in MPA of the healthy one.

**Figure 9:**
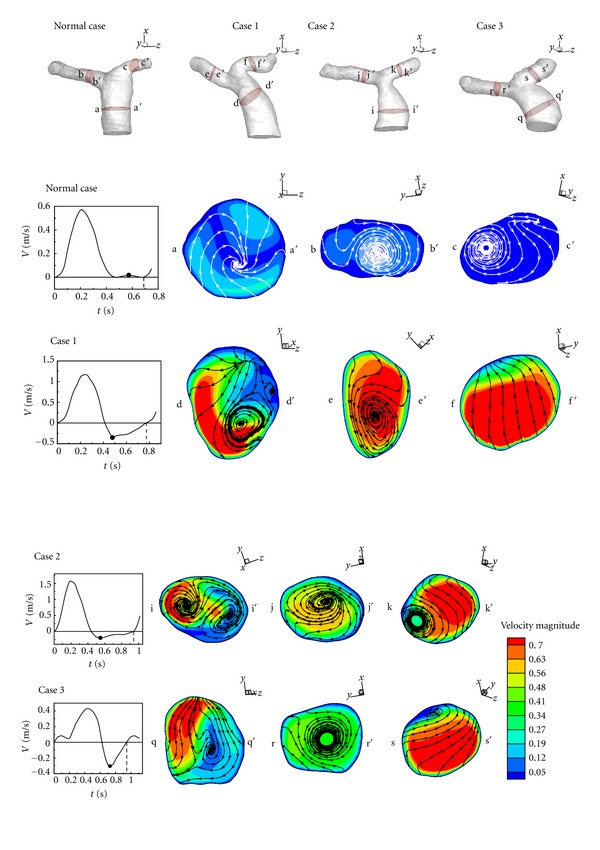
Flow patterns and velocity distribution in minimum of a cardiac cycle.

Since vortices are found in MPA of Cases  1–3 in regurgitation, it is interesting to investigate the flow structure in specified cross-sections.  Figure 9 shows flow patterns of the healthy one and Cases  1–3 in specified cross-sections of MPA, LPA, and RPA in diastole. According to Perry and Steiner's [30] definitions of larger vortex in 3D flow, a stable node is found in the cross-section a-a′ of the healthy MPA. Stable focuses appear in cross-sections b-b′ and c-c′ of healthy RPA and LPA. The stable node in healthy does not occur in Cases  1–3. One or more focuses are found in MPA of Cases  1–3. An unstable bifurcation line, a saddle point, and a stable focus happen in the cross-section d-d′ of MPA of Case 1. A stable focus and another unstable focus are observed in the cross-section i-i′ of MPA of Case 2. In Case  3, there are a saddle point and a stable focus presenting in the cross-section q-q′ of MPA. In addition, the focus appearing in the healthy RPA is found in Cases  1–3. It is stable in the healthy one and Cases 1 and 3, but it is unstable in Case 2. The focus in healthy LPA does not appear in all Cases  1–3. The flow pattern in LPA of Case 1 shows a very strong and smooth and reversed flow. In Cases 2 and 3, the focus shrinks because of the strong reversed flow.

**Figure 10:**
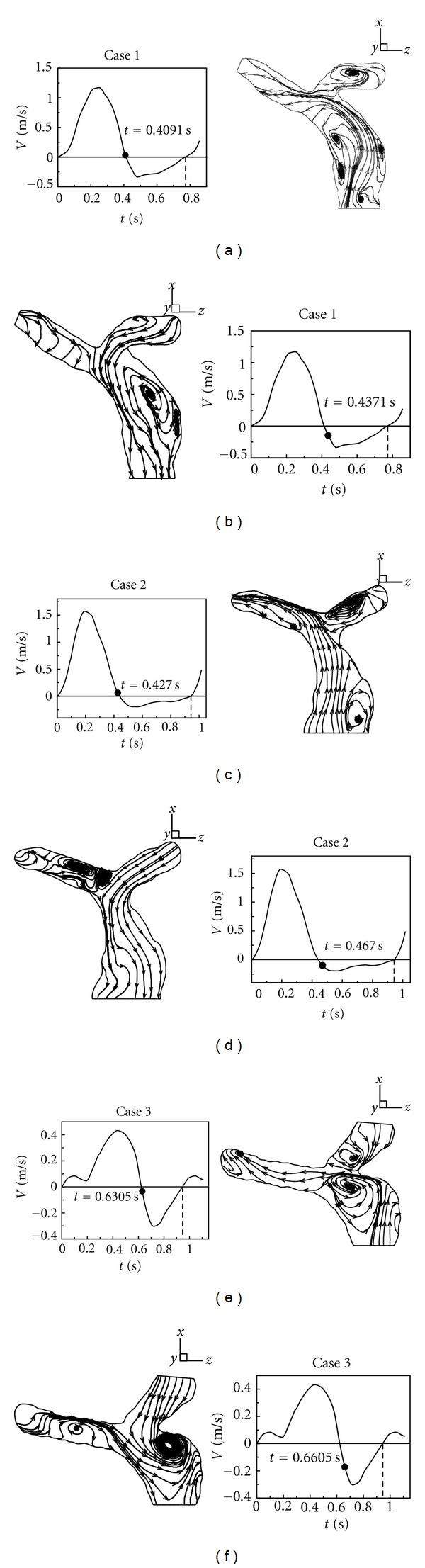
First time of regurgitation happening in LPA and RPA.

In order to know whether regurgitation happens first in LPA or not, Figure 10 exhibits the time of inception of regurgitation in RPA and LPA in Cases  1–3. Figures 10(a), 10(c), and 10(e) present that regurgitation first occurs in LPA of Cases  1–3. Also, an obvious recirculation appears in LPA of Cases  1–3. In terms of Figures 10(b), 10(d), and 10(f), it turns out that the time of inception of regurgitation in RPA is nearly 0.03 seconds behind that in LPA of Cases  1–3.

According to Figures 6, 7, and 8, the complex flow patterns always happen in regurgitation of MPA of Cases  1–3. 3-D strong vortex motion is found in this stage as shown in Figures 7 (1-4), (2-4), and (3-4) and 8 (1-6), (2-6), and (3-6) in the ends of systole and diastole because the direction of blood flow in MPA changes. The 3D vortex motion in MPA may cause extraload in the right ventricle since the healthy case does not have regurgitation and the vortex motion.

In clinical cases, there is no dilation in MPA before repair of TOF. The dilation often occurs in MPA after repair of TOF. In terms of numerical results, it is found that 3-D strong vortex motion appears in dilation of MPA in regurgitation. Therefore, the dilation causes abnormal flow distribution in MPA during regurgitation period in a cardiac cycle. Apparently, the dilation in MPA is an important factor to influence variation of blood flow in diastole.

### 3.2. Analysis of Pressure Distribution

**Figure 11:**
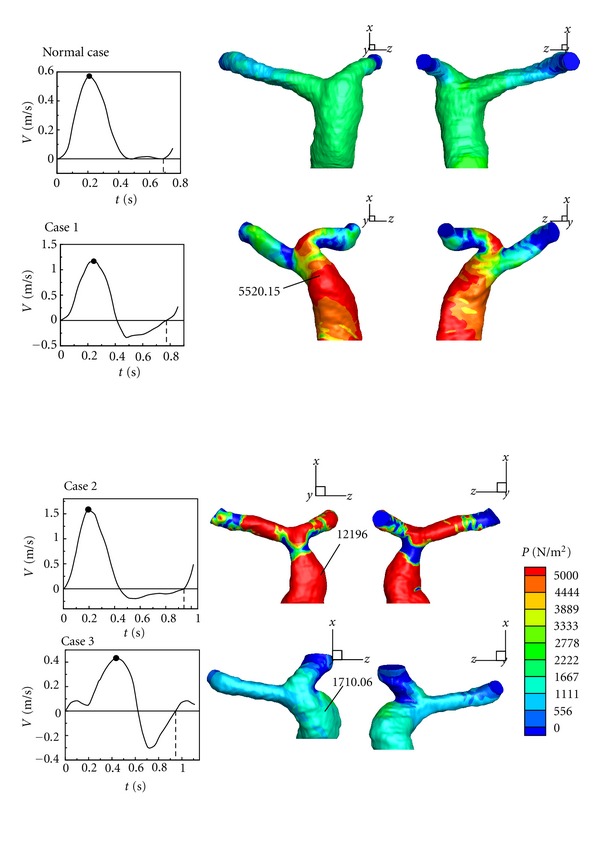
Comparing the distribution of pressure in pulmonary arteries in maximum of a cardiac cycle.

**Figure 12:**
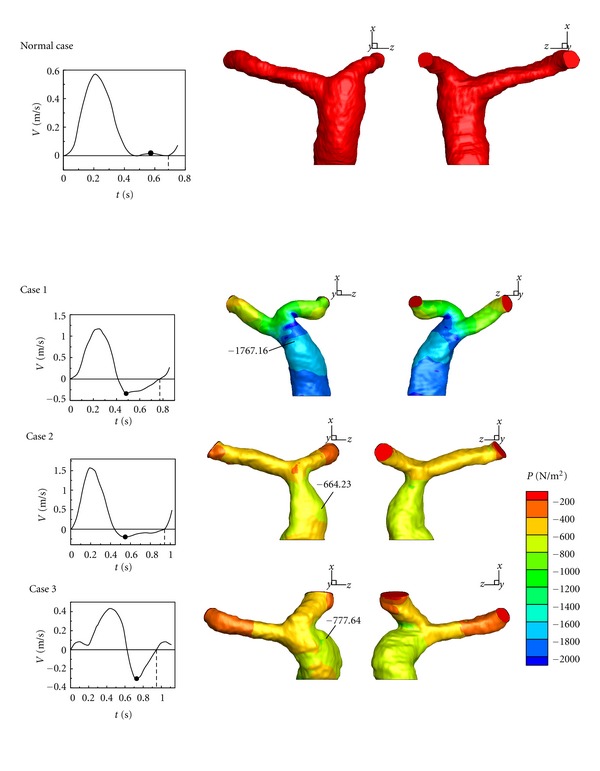
Comparing the distribution of pressure in pulmonary arteries in minimum of a cardia cycle.

In our models, the dilation occurs in MPA of all repaired TOF cases. However, the reason causing dilation is not clear. Therefore, it is necessary to investigate the dilated area. This section gives the analysis of the pressure distribution in pulmonary arteries in a cardiac cycle.  Figure 11 shows pressure distributions of the healthy case and Cases  1–3 in the maximum of a cardiac cycle. A high pressure region is found in the bifurcation area for all cases. In addition, another high pressure region is observed in the dilation of MPA of Cases  1–3. Pressures distributions of Cases 1 and 2 are very nonuniform. It should be noticed that there is a stenosis between the bifurcation and MPA in Case 2, so the high pressure in the dilation of Case 2 is extreme and higher than other cases.  Figure 12 displays pressure distributions of the healthy one and Cases  1–3 in diastole. A low pressure region is discovered in the dilation area of MPA in Cases  1–3. It is caused by the 3-D vortex motion as shown in Figure 8. According to Figures 11 and 12, the pressure varies extremely in the dilation of MPA in a cardiac cycle. For example, the pressure change in the dilation of Case 2 is around 12860 N/m^2^. Nevertheless, the maximum pressure change in the healthy one is around 2100 N/m^2^. The large pressure variation in MPA would affect the vessels wall in MPA after repaired TOF seriously.

In terms of Figures 11 and 12, large pressure change may cause obvious deformation of MPA. As we mentioned, the 3-D vortex motion due to regurgitation in MPA plays an important role in the low pressure region. The situation could lead to deterioration of dilation in the longer term.

**Figure 13:**
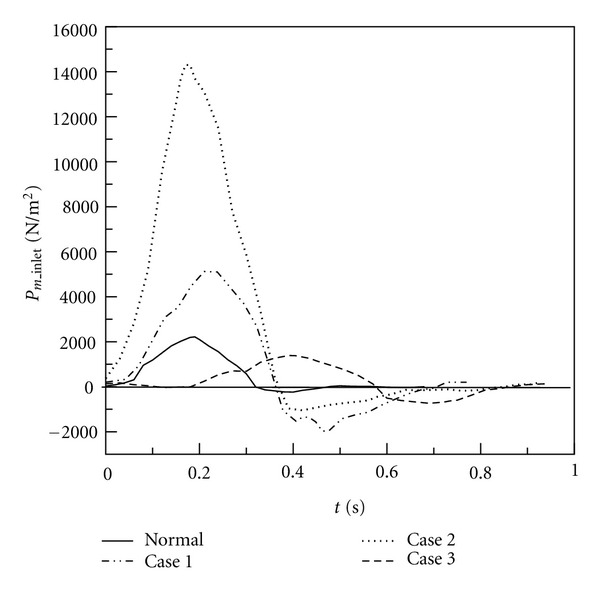
Time histories of inlet pressure of MPA in a cardiac cycle.

Figure 13 presents time histories of inlet pressure of MPA of Cases  1–3 and the healthy one in a cardiac cycle. The solid curve presents the inlet pressure of the healthy one. The peak value of pressure is around 2000 N/m^2^ in systole. Very low negative pressure occurs in diastole. Dashed curves present pressure variations of Cases  1–3. It is found that the peak values of pressure of Cases 1 and 2 are higher than the healthy one in systole. Moreover, negative pressure happens in Cases 1 and 2 in the diastole since regurgitation happens. It is found that the extreme prssure variation exists at the inlet of MPA of Cases 1 and 2. The cardiac cycle of Case  3 is longer than other cases, so the systole period of Case  3 is longer than other cases as well. Due to the larger inlet cross-section of Case  3 than other cases as shown in Table 3, the peak value of inlet pressure of Case  3 is not so large as Cases 1 and 2. However, obvious negative pressure due to regurgitation exists in Case  3 in diastole. It is known that the damage of the semilunar valve is one of the complications in patients after repaired TOF. Large inlet pressure variations shown in Cases 1 and 2 may be one of the reasons to induce dysfunction of the pulmonary valve.

### 3.3. Mass Flow Rate and Regurgitation

**Figure 14:**
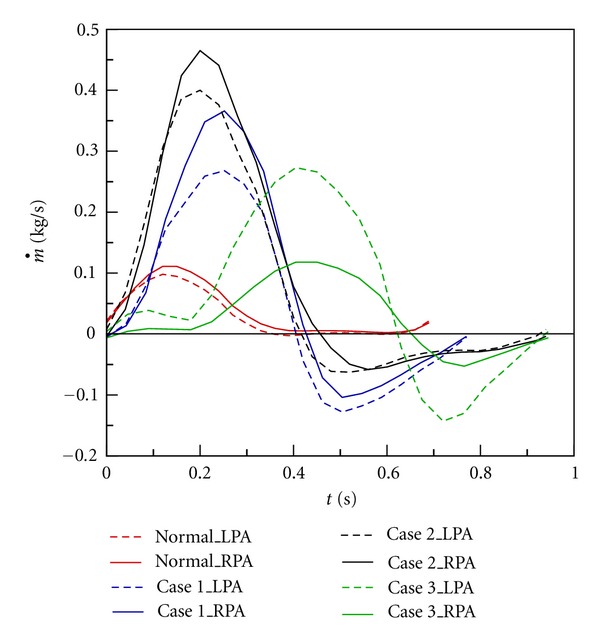
Time histories of mass flow rate in a cardiac cycle.

The pulmonary artery geometry effects the mass flow rate distributions in MPA, LPA and RPA.  Figure 14 shows time histories of mass flow rates in LPA and RPA of Cases  1–3 in a cardiac cycle. Solid and dashed curves represent mass flow rates in RPA and LPA, respectively. Red curves show the variations of mass flow rates in the healthy one. The mass flow rates in LPA and RPA are very close in the healthy one. There is no regurgitation in LPA and RPA. Blue, black, and green curves represent mass flow rates in Cases  1–3. Serious regurgitation is found in RPA and LPA of Cases  1–3 in diastole. Also, it is found that regurgitation always appears in LPA first in three cases. The forward flow amount of LPA is less than RPA in Cases 1 and 2. The phenomenon does not happen in Case  3. It may be because the diameter of LPA is larger than that of RPA as shown in Table 3. Meanwhile, the amount of reversed flow in LPA is larger than RPA in Cases  1–3. Table 3 presents regurgitation volumes of RPA and LPA of Cases  1–3 in a cardiac cycle. The ratio of regurgitation of LPA to RPA is always higher than 1. Diameters of RPA and LPA of Cases 1 and 2 are close to each other (see Table 2). Nevertheless, the ratio of *θ*_1_ to *θ*_2_ of Case 1 is larger than that of Case 2. As a result, the ratio of regurgitation of LPA to RPA in Case 1 is higher than Case 2.

### 3.4. Comparison between Numerical Results and Clinical PC-MR Measurement Data

**Figure 15:**
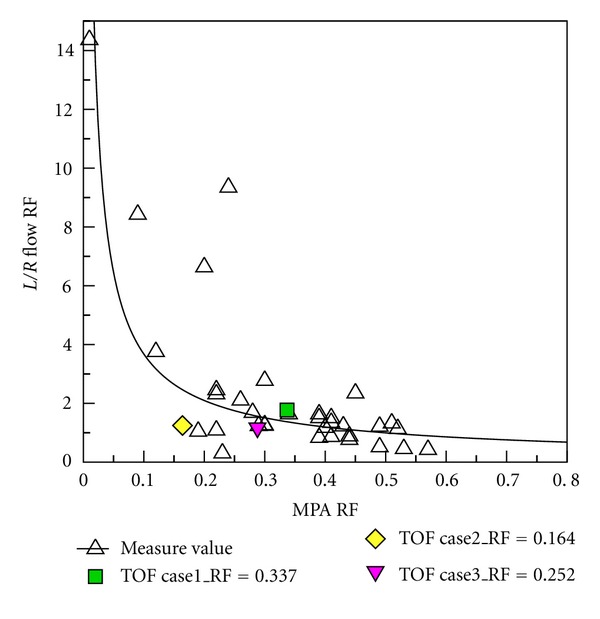
Comparing the L/R RF and MPA RF relationship between medical measurement and numerical computational values.

**Figure 16:**
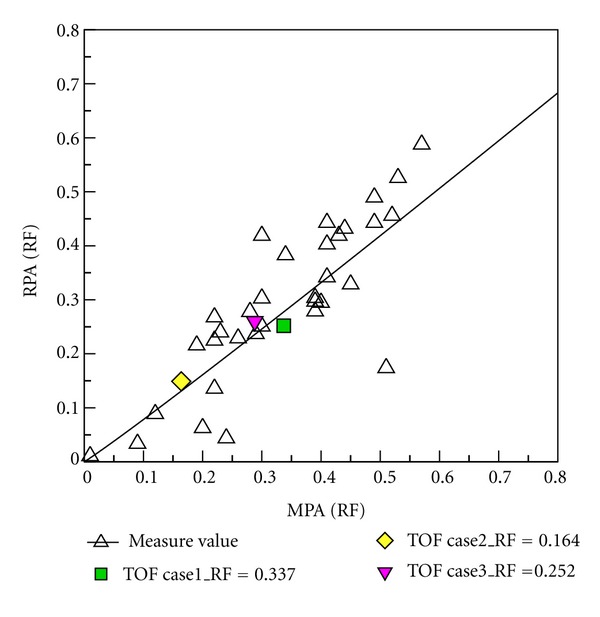
Comparing the RPA RF and MPA RF relationship between medical measurement and numerical computational values.

**Figure 17:**
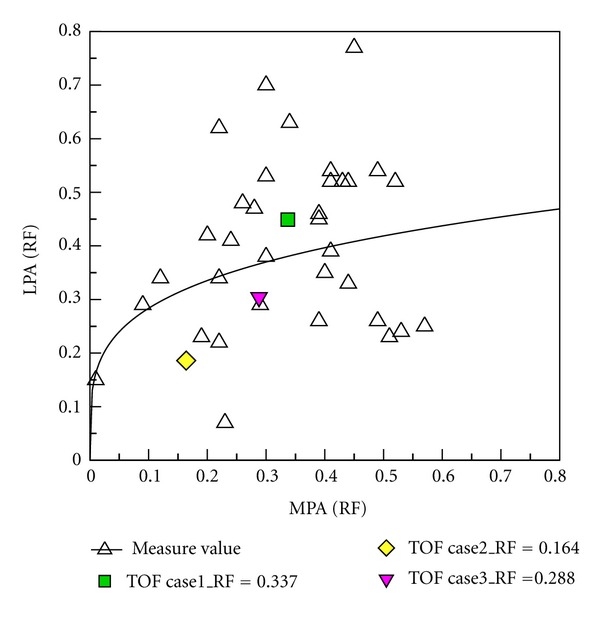
Comparing the LPA RF and MPA RF relationship between medical measurement and numerical computational values.

Clinical PC MR measurement data from 34 patients after repair of TOF were provided by Kaohsiung Veterans General Hospital. Regurgitation in MPA, LPA, and RPA was determined in PC MR measurements. Subsequently, the numerical results are compared with those measurement data.  Figure 15 shows that RF of MPA varies from 0.1 to 0.6. Measurement data and numerical results are denoted as black hollow and color symbols, respectively. The ratio of LPA RF to RPA RF decreases as RF of MPA increases in measurement data. It is found that numerical results are close to measurement data.  Figure 16 shows the relationship between regurgitation of RPA and MPA. Essentially, the regurgitation of RPA is proportional to that of MPA in measurement data. The numerical results also show the trend in Figure 16.  Figure 17 reveals the relationship between LPA RF and MPA RF. Measurement data are scattered in the figure. Predicted results are within measurement data as shown in the figure. Consequently, the numerical computational values are acceptable based on RF analysis with clinical measurement values as shown in Figures 15, 16, and 17.

## 4. Conclusions

We utilize peripheric data of pulmonary arteries measured by MRI to reconstruct 3-D models of pulmonary arteries of healthy person and patients after repair of TOF. Those reconstructed models of three real pulmonary arteries are used to simulate blood flow motion. The degree of pulmonary regurgitation and geometry of pulmonary artery have significant effects on the blood flow variation.

Flow patterns are visualized by streamlines and velocity magnitude in numerical results. The recirculation region is found in LPA of all pulmonary arteries after repair of TOF in acceleration of systole. It obstructs the blood flow toward the left lung. The strong vortices are found in the dilation area of MPA in repaired pulmonary arteries during diastole of a cardiac cycle. Nevertheless, there is no such vortex in healthy MPA in diastole. In the ends of systole and diastole, 3-D complex vortices motion appears in repaired TOF pulmonary arteries. The blood flow of the health one is smoother than those. According to numerical results, it is found that the dilation causes abnormal flow distribution in MPA during regurgitation period in a cardiac cycle.

The comparison of the flow patterns and regurgitation proves that regurgitation happens first in LPA. The amount of regurgitation in LPA is larger than RPA in numerical results. The effect of predicted pressure distribution is also discussed. The dilation has extreme pressure change in a cardiac cycle. Nevertheless, the health MPA does not have such extreme pressure variation. The high pressure is one of the important factors to generate pulmonary arteries dilation.

We could understand the influence of pulmonary regurgitation and the blood flow patterns through this study. The results will be a useful reference for medical doctors before they perform operations for TOF patients.

## Figures and Tables

**Table 1:** Parameters of pulmonary arteries.

Case	Normal	Case 1	Case 2	Case 3
*L*_RPA_ (cm)	5.23	3.99	4.93	5.27
*L*_LPA_ (cm)	2.71	3.60	2.36	2.39
*L*_MPA_ (cm)	3.85	5.31	4.17	3.14
*d*_RPA_ (cm)	1.39	1.40	1.13	1.21
*d*_LPA_ (cm)	1.36	1.30	1.09	1.68
*d*_LPA_/*d*_LPA_	0.98	0.92	0.96	1.39
*θ*_1_ (degree)	125°	134°	106°	136°
*θ*_2_ (degree)	112°	52°	83°	70°
*θ*_1_/*θ*_2_	1.12	2.58	1.28	1.94
*A*_Inlet_ (cm^2^)	3.48	4.58	4.89	5.83

**Table 2:** The regurgitation fraction (RF).

Case		RF	
	MPA	LPA	RPA
1	0.337	0.449	0.252
2	0.164	0.186	0.149
3	0.288	0.304	0.260

**Table 3:** Regurgitation volume (cm^3^) in a cardiac cycle.

Case	Regurgitation volume			LPA/RPA
	MPA	LPA	RPA
1	47	27	20	1.35
2	32	17	15	1.1
3	33	24	9	2.67

## Nomenclatures

### Latin Symbols

$\dot{m}$: Mass flow rate, kg·s^−1^
*P*: Pressure, Pa
*Q*_*b*_: Backward blood flow volume in a cardiac cycle, m^3^
*Q*_*f*_: Forward blood flow volume in a cardiac cycle, m^3^
RF: Regurgitation fraction
*t*: Time, s
**u**: Velocity vector, m·s^−1^.

### Greek Symbols

*θ*: Bifurcation angle, degree
*μ*: Viscosity, kg·m^−1^·s^−1^
*ν*: Kinematic viscosity, m^2^·s^−1^
*ρ*: Density, kg·m^−3^.
